# Supplementary material for: Thiamine-Mediated Microbial Interaction between Auxotrophic Rhodococcus ruber ZM07 and Prototrophic Cooperators in the Tetrahydrofuran-Degrading Microbial Community H-1
Source: Microbiol Spectr. 2023 May 1;11(3):e04541-22. doi: 10.1128/spectrum.04541-22 (PMC10269752; doi:10.1128/spectrum.04541-22)
Supplement: Supplemental file 1 — Supplemental material. Download spectrum.04541-22-s0001.pdf, PDF file, 3.4 MB [file spectrum.04541-22-s0001.pdf]

# Supplemental Material

# Thiamine-mediated microbial interaction between auxotrophic *Rhodococcus ruber* ZM07 and prototrophic cooperators in a tetrahydrofuran-degrading microbial community H-1

**Hui Huang[1,2], Hao Wu[1], Minbo Qi[1], Haixia Wang[1], Zhenmei Lu[1*]**

[1]MOE Laboratory of Biosystem Homeostasis and Protection, College of Life Sciences, Zhejiang University, Hangzhou, 310058, China

[2]Institute of Translational Medicine, Zhejiang University, Hangzhou, 310029, China

*Corresponding author: Zhenmei Lu, Ph. D, MOE Laboratory of Biosystem Homeostasis and Protection, College of Life Sciences, Zhejiang University, Hangzhou, Zhejiang, 310058, China

Phone: +86-571-88206279, Fax: +86-571-88206485, Email: lzhenmei@zju.edu.cn

## SUPPLEMENTAL METHODS

**Chemicals.** Tetrahydrofuran (THF, ≥ 99.9% purity), and thiamine (vitamin B1, 98% purity) were purchased from Aladdin Holdings Group Co., Ltd. (Shanghai, China). Other chemicals were either purchased from Solarbio Science & Technology Co., Ltd. (Beijing, China) or Aladdin Holdings Group Co., Ltd. (Shanghai, China).

**Microbial medium.** One liter of basal salt medium (BSM) (1) contains 3.240 g $K_2HPO_4$, 1.000 g $NaH_2PO_4 \cdot H_2O$, 2.000 g $NH_4Cl$, 0.123 g $C_6H_8NNa_3O_7$, 0.200 g $MgSO_4 \cdot 7H_2O$, 0.012 g $FeSO_4 \cdot 7H_2O$, 0.003 g $MnSO_4 \cdot H_2O$, 0.003 g $ZnSO_4 \cdot 7H_2O$, and 0.001 g $CoCl_2 \cdot 6H_2O$. One liter of the pantothenate medium (2) using to isolate strain ZM11 contains 0.27 g $KH_2PO_4$, 0.26 g $MgSO_4 \cdot 7H_2O$, 0.28 g $FeSO_4 \cdot 7H_2O$, 0.15 g $MnSO_4$, and 0.21 g $Na_2MoO_4$, 2.57 g potassium pantothenate and 15 g agar. pH was adjusted to 7.0 with KOH. One liter of the R2A agar (3, 4) using to isolate strain ZM12, contains 0.5 g yeast extract, 0.5 g proteose peptone, 0.5 g casamino acids, 0.5 g glucose, 0.5 g soluble starch, 0.3 g $K_2HPO_4$, 0.05 g $MgSO_4 \cdot 7H_2O$, 0.3 g sodium pyruvate, and 15 g agar. pH was adjusted to 7.2 with $K_2HPO_4$.

**cDNA library construction, RNA-seq and data analysis.** In order to obtain pure RNA, genomic DNA was removed from total RNA using RNase-Free DNase Set (Qiagen, Germany) and then concentrated using RNeasy MinElute Cleanup Kit (Qiagen, Germany). The concentrated RNA was converted to double-stranded cDNA using the SuperScript® III First-Strand Synthesis System (Invitrogen, USA) with priming via random hexamers for first-stranded synthesis, and the SuperScript® Double-Stranded cDNA Synthesis Kit (Invitrogen, USA) for second-stranded synthesis. The concentration and purity of total DNA and synthetic double-stranded cDNA were checked with a NanoDrop 2000c spectrophotometer (Thermo Scientific, USA). All above kits were used according to the manufacturer's instructions. Metatranscriptomic libraries were constructed according to the NEBNext® UltraTM RNA Library Prep Kit (Illumina, New England Biolabs), and were then sequenced by Novogene Bioinformatics Technology Co., Ltd. (Beijing, China) on an Illumina NovaSeq 6000 platform (Illumina).

Raw data were processed by removing the rRNA sequences and low-quality reads (the base number Qphred ≤ 20 accounts for more than 50% of the read length). Trinity (version 2.1.1) was

employed for de novo transcriptomic assemblies. The readcount data obtained from gene expression analysis were further used for gene differential expression analysis. FPKM (fragments per kilobase of transcripts permappedmillion fragmentsmethod) was used to analyze the gene expression according to read count data (5). Then the significantly differentially expressed genes (DEGs) between different two groups (T1-THI vs T1-CK, T40-CK vs T1-CK, T40-THI vs T1-THI, and T40-CK vs T1-CK) were identified using the p values (6). A false discovery rate (FDR) $< 0.05$ and FPKM ratio $\geq 2$ was considered significant. All the gene sequences were searched against the Kyoto Encyclopedia of Genes and Genomes (KEGG) database (7) to obtain functional annotation information. The significantly enriched KEGG pathways in the DEGs were identified with the hypergeometric test in the entire genome background. The calculated $p$ value was corrected by the Bonferroni method with a threshold of $p$ value $\leq 0.05$ to define KEGG pathways as significantly enriched. All the statistical analyses and graphics were executed using Microsoft® Excel and R software (version 3.3.2).

## SUPPLEMENTAL TABLES

**Table S1** The samples' information for high-throughput sequencing.

| Groups | Samples' information | Independent replicate numbers of 16S rRNA gene sequencing | Independent replicate numbers of Metatranscriptome |
|---|---|---|---|
| T1-THI | Microbial community H-1 was collected at 72 h in the $1^{st}$ transfer with exogenous thiamine | 12 | 3 |
| T1-CK | Microbial community H-1 was collected at 72 h in the $1^{st}$ transfer without exogenous thiamine | 12 | 3 |
| T40-THI | Microbial community H-1 was collected at 72 h in the $40^{th}$ transfer with exogenous thiamine | 12 | 3 |
| T40-CK | Microbial community H-1 was collected at 72 h in the $40^{th}$ transfer without exogenous thiamine | 12 | 3 |

**Table S2** Primers used to quantify strains ZM07, ZM11, and ZM12 using qPCR in this study.

| Primers | Sequence 5’ - 3’ |
|---|---|
| ZM07-F | GAACACGCTCGCCGAGAACTCC |
| ZM07-R | GTTCGTCAGCAGCAGCCCGTAG |
| ZM11-F | GGTGCAATCGTTCCAGCGGTCT |
| ZM11-R | CTGCGCGAAGTGCGGTCGAT |
| ZM12-F | CAAGCAACTGGCAGACAAGTTCA |
| ZM12-R | GCCCGAAGAAGTACGGGTTGCA |

**Table S3** Changes of the genes responsible for thiamine metabolism between T1-THI and T1-CK.

| Genes | Gene/protein annotation | $log_2$FoldChange |
|---|---|---|
| **Other bacteria except ZM07** | | |
| *Dxs* | 1-Deoxy-D-xylulose-5-phosphate synthase | -1.190 |
| *ThiC* | Phosphomethylpyrimidine synthase ThiC | -4.660 |
| *IscS* | Cysteine desulfurase | -1.235 |
| *ThiM* | Hydroxyethylthiazole kinase | -4.155 |
| *TenA* | Thiaminase II | -3.026 |
| *ThiP* | Thiamine/thiamine pyrophosphate ABC transporter, permease protein | -4.788 |
| *ThiY* | Hypothetical protein | -4.906 |
| **ZM07** | | |
| *ThiL* | Thiamine-monophosphate kinase | 2.283 |

**Table S4** Changes of the genes responsible for THF degradation in *Rhodococcus ruber* ZM07.

| **Genes** | **Gene/protein annotation** | **$log_2$FoldChange** | |
|---|---|---|---|
| | | T1-THI vs T1-CK | T40-THI vs T40-CK |
| *Thm* | Gene cluster of THF degradation | 2.545 | 3.155 |
| *Ndh* | NADH dehydrogenase | 1.702 | 3.301 |
| *NuoN* | NADH-quinone oxidoreductase subunit N | 2.113 | 2.081 |
| *NuoG* | NADH-quinone oxidoreductase subunit G | 1.611 | 1.643 |
| *CoxA* | Cytochrome c oxidase subunit I | 2.024 | 1.725 |
| *ResB, ccs1* | Cytochrome c biogenesis protein | 2.658 | 2.366 |

**Table S5** Up/down regulated genes in *Rhodococcus ruber* ZM07 / *Hydrogenophaga intermedia* ZM11.

| Numbers | Ko_name | *Rhodococcus ruber* ZM07 | | *Hydrogenophaga intermedia* ZM11 | | NR_description |
|---|---|---|---|---|---|---|
| | | $log_2$FoldChange | *p* value | $log_2$FoldChange | *p* value | |
| 1 | MUT | 2.960 | 1.157E-13 | 3.204 | 2.108E-08 | Methylmalonyl-CoA mutase |
| 2 | gabD | 4.345 | 1.412E-21 | -1.329 | 9.284E-08 | Dehydrogenase |
| 3 | glnA, GLUL | 3.784 | 6.522E-15 | -1.448 | 8.910E-05 | Glutamine synthetase |
| 4 | ACSS, acs | 3.354 | 4.726E-13 | -1.511 | 8.314E-10 | AMP-dependent synthetase |
| 5 | OPLAH, OXP1, oplAH | 3.280 | 3.735E-15 | -1.011 | 1.918E-04 | 5-Oxoprolinase |
| 6 | E3.6.3.- | 3.254 | 2.300E-12 | -1.837 | 2.664E-14 | ATP-dependent transcriptional regulator |
| 7 | hrpA | 3.161 | 9.361E-11 | -1.023 | 5.651E-06 | ATP-dependent RNA helicase HrpA |
| 8 | recG | 3.049 | 1.337E-10 | -1.950 | 5.422E-14 | ATP-dependent DNA helicase RecG |
| 9 | lepA | 2.990 | 2.780E-10 | -1.168 | 5.390E-05 | Required for accurate and efficient protein synthesis under certain stress conditions. |
| 10 | ABC-2.A | 2.951 | 2.313E-09 | -1.626 | 7.981E-11 | Triacylglycerol lipase |
| 11 | DPO3A1, dnaE | 2.926 | 1.203E-09 | -1.315 | 2.040E-09 | DNA polymerase III (alpha subunit) |
| 12 | dnaE2 | 2.902 | 3.762E-10 | -1.852 | 4.826E-14 | Error-prone DNA polymerase |
| 13 | gyrA | 2.881 | 4.037E-13 | -1.310 | 1.176E-08 | DNA gyrase negatively supercoils closed circular double-stranded DNA in an ATP-dependent manner |
| 14 | vanA | 2.821 | 5.664E-10 | -1.036 | 9.336E-06 | Rieske (2fe-2S) domain-containing protein |
| 15 | mfd | 2.819 | 1.461E-08 | -1.119 | 6.363E-07 | ATP-dependent DNA helicase (RecG) |

| Numbers | Ko_name | *Rhodococcus ruber* ZM07 | | *Hydrogenophaga intermedia* ZM11 | | NR_description |
|---|---|---|---|---|---|---|
| 16 | ftsK, spoIIIE | 2.802 | 1.065E-09 | -1.265 | 2.053E-08 | Cell division protein FtsK |
| 17 | phbC, phaC | 2.799 | 4.437E-09 | -1.592 | 7.940E-11 | Poly(r)-hydroxyalkanoic acid synthase, class |
| 18 | SARS, serS | 2.764 | 1.807E-08 | -1.543 | 4.207E-08 | MFS transporter |
| 19 | uvrA | 2.742 | 9.337E-09 | -1.156 | 8.254E-07 | The UvrABC repair system catalyzes the recognition and processing of DNA lesions. |
| 20 | livK | 2.739 | 9.664E-07 | -1.633 | 1.192E-09 | ABC transporter permease |
| 21 | tgt, QTRT1 | 2.717 | 1.884E-08 | -1.002 | 1.213E-04 | tRNA guanosine(34) transglycosylase Tgt |
| 22 | purL, PFAS | 2.690 | 9.888E-09 | -1.358 | 1.705E-07 | Phosphoribosylformylglycinamidine synthase |
| 23 | K07161 | 2.677 | 1.281E-08 | -1.368 | 4.108E-08 | VWA domain-containing protein |
| 24 | copA, ATP7 | 2.667 | 2.966E-08 | -1.365 | 2.812E-08 | P-type ATPase |
| 25 | E1.14.13.1 | 2.652 | 6.666E-06 | -1.435 | 5.440E-09 | 3-Hydroxybenzoate 6-hydroxylase |
| 26 | E1.3.99.- | 2.630 | 3.183E-03 | -1.128 | 2.176E-05 | Acyl-CoA dehydrogenase |
| 27 | ABC.CD.P | 2.629 | 2.881E-08 | -1.284 | 3.812E-09 | ABC transporter (permease) |
| 28 | E5.1.99.4, AMACR, mcr | 2.621 | 1.547E-07 | -1.129 | 6.176E-07 | L-carnitine dehydratase bile acid-inducible protein F |
| 29 | murJ, mviN | 2.611 | 7.267E-09 | -1.117 | 1.061E-07 | Integral membrane protein MviN |
| 30 | pps, ppsA | 2.607 | 5.245E-08 | -1.322 | 3.782E-10 | Pyruvate phosphate dikinase |
| 31 | xseA | 2.585 | 3.052E-08 | -1.184 | 1.231E-08 | Bidirectionally degrades single-stranded DNA into large acid-insoluble oligonucleotides |
| 32 | DARS, aspS | 2.581 | 1.749E-08 | -1.237 | 8.213E-06 | Aspartyl-trna synthetase |
| 33 | IARS, ileS | 2.577 | 7.854E-08 | -1.146 | 1.483E-08 | Amino acids such as valine, to avoid such errors it has two additional distinct tRNA(Ile)-dependent editing activities. |
| 34 | ABCB-BAC | 2.518 | 4.619E-10 | -1.023 | 4.628E-06 | Hypothetical protein |

| Numbers | Ko_name | *Rhodococcus ruber* ZM07 | | *Hydrogenophaga intermedia* ZM11 | | NR_description |
|---|---|---|---|---|---|---|
| 35 | NADSYN1, QNS1, nadE | 2.483 | 2.594E-08 | -1.058 | 6.640E-07 | Nitrilase cyanide hydratase and apolipoprotein n-acyltransferase |
| 36 | E4.2.1.2A, fumA, fumB | 2.462 | 2.731E-08 | -1.155 | 1.800E-08 | Fumarate |
| 37 | atoB | 2.442 | 8.352E-09 | -1.085 | 2.197E-06 | Acetyl-coa acetyltransferase |
| 38 | ilvD | 2.377 | 1.231E-07 | -1.215 | 1.882E-08 | Dihydroxy-acid dehydratase |
| 39 | E3.5.1.28B, amiA, amiB, amiC | 2.363 | 1.633E-08 | -1.233 | 3.098E-08 | N-acetylmuramoyl-l-alanine amidase (EC 3.5.1.28) |
| 40 | AARS, alaS | 2.331 | 2.753E-07 | -1.572 | 4.624E-06 | Alanine--tRNA ligase |
| 41 | ACSL, fadD | 2.292 | 2.873E-07 | -1.019 | 1.546E-06 | AMP-dependent synthetase |
| 42 | livF | 2.259 | 3.437E-04 | -1.982 | 2.280E-08 | (ABC) transporter |
| 43 | murC | 2.259 | 2.655E-06 | -1.018 | 9.920E-07 | Peptidase S41 |
| 44 | uup | 2.241 | 2.539E-06 | -1.306 | 9.931E-06 | ABC transporter |
| 45 | PARS, proS | 2.200 | 2.437E-06 | -1.204 | 1.084E-07 | Catalyzes the attachment of proline to tRNA (Pro) in a two-step reaction proline is first activated by ATP to form Pro - AMP and then transferred to the acceptor end of tRNA (Pro). |
| 46 | E6.4.1.4B | 2.156 | 7.240E-06 | -1.210 | 1.922E-07 | Carboxylase |
| 47 | coaBC, dfp | 2.079 | 7.116E-06 | -1.260 | 2.136E-08 | Phosphopantothenoylcysteine decarboxylase |
| 48 | tktA, tktB | 2.071 | 3.741E-06 | -1.294 | 1.799E-07 | Transketolase |
| 49 | ggt | 2.059 | 6.358E-05 | -1.028 | 4.723E-07 | Gamma-glutamyltranspeptidase (EC 2.3.2.2) |
| 50 | narG | 2.051 | 1.009E-04 | -1.427 | 5.209E-07 | Nitrate reductase, alpha subunit |
| 51 | metE | 2.003 | 2.587E-07 | -1.202 | 6.836E-09 | Methionine synthase |
| 52 | K00666 | 1.988 | 3.640E-04 | -1.624 | 8.294E-11 | AmP-dependent synthetase and ligase |

| Numbers | Ko_name | *Rhodococcus ruber* ZM07 | | *Hydrogenophaga intermedia* ZM11 | | NR_description |
|---|---|---|---|---|---|---|
| 53 | ENO, eno | 1.962 | 1.700E-05 | -1.259 | 7.671E-08 | Catalyzes the reversible conversion of 2- phosphoglycerate into phosphoenolpyruvate. |
| 54 | ABC.PE.S | 1.843 | 1.100E-03 | -1.486 | 2.233E-07 | Peptide ABC transporter permease |
| 55 | E1.2.1.3 | 1.832 | 7.784E-05 | -1.043 | 1.303E-04 | Aldehyde dehydrogenase |
| 56 | E2.2.1.6L, ilvB, ilvG, ilvI | 1.765 | 4.990E-05 | -1.531 | 4.098E-12 | Acetolactate synthase |
| 57 | aceA | 1.618 | 1.931E-03 | -1.020 | 1.391E-06 | Oxygen-independent coproporphyrinogen III oxidase |
| 58 | PTS-EI.PTSI, ptsI | 1.485 | 1.452E-03 | -1.233 | 3.995E-09 | General (non-sugar-specific) component of the phosphoenolpyruvate-dependent sugar phosphotransferase system (sugar PTS) |

## SUPPLEMENTAL FIGURES

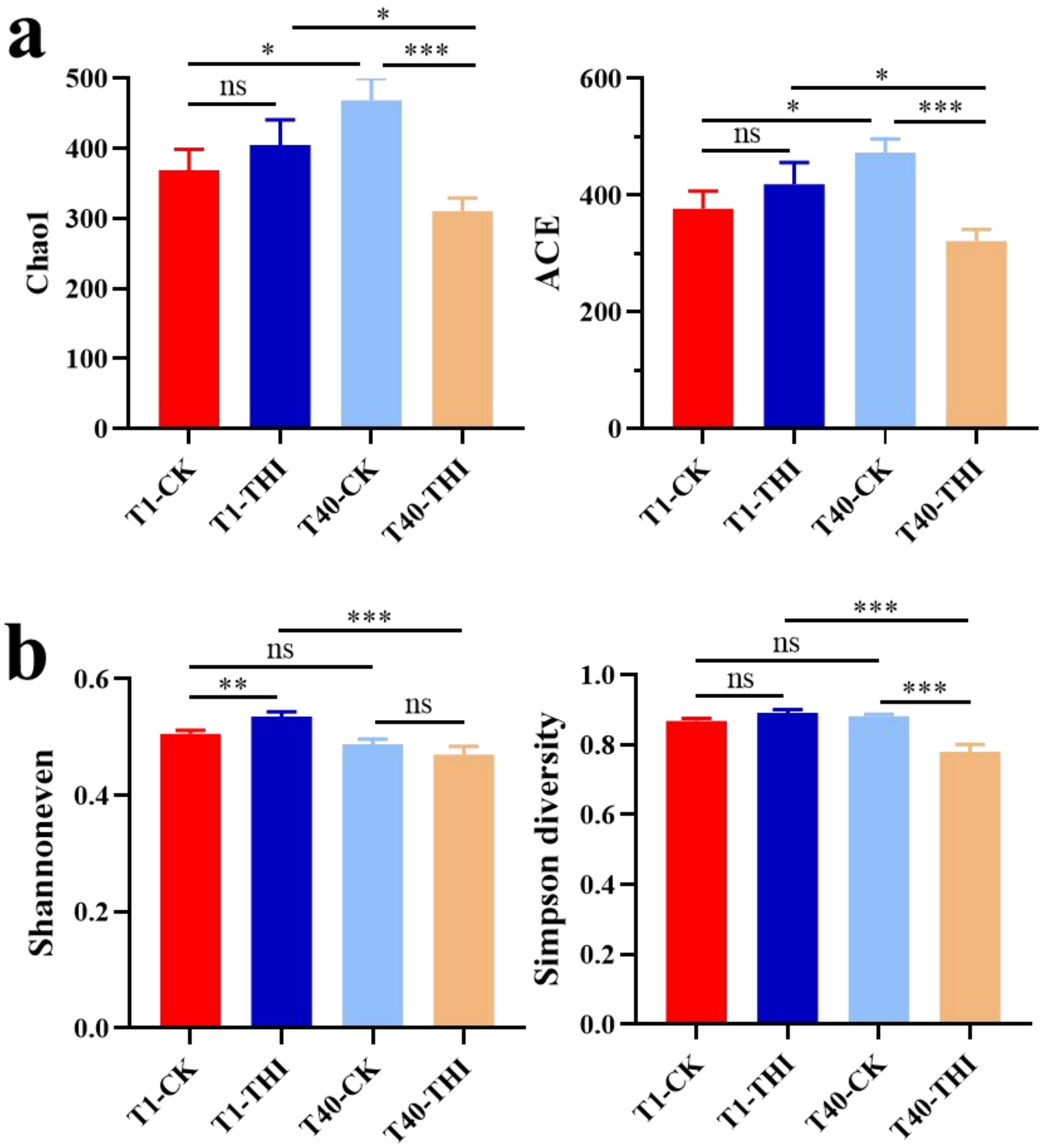


**Figure S1** The α-diversity of the microbial community H-1 in different groups. (a) Chao1 and ACE of OTUs show the community richness. (b) The Shannon and Simpson diversity of OTUs show the community diversity. The $p$ value indicates statistical significance determined using Student's $t$-test (n = 12, ns > 0.05, *$p < 0.05$, **$p < 0.01$, ***$p < 0.001$). Error bars represent the standard error of the mean.

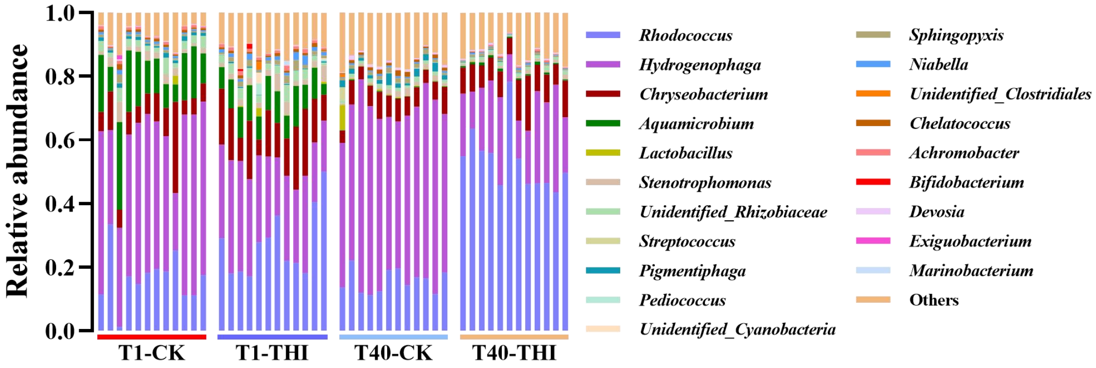


**Figure S2** Community histogram of all samples showing the microbial composition profiling at the genus level.

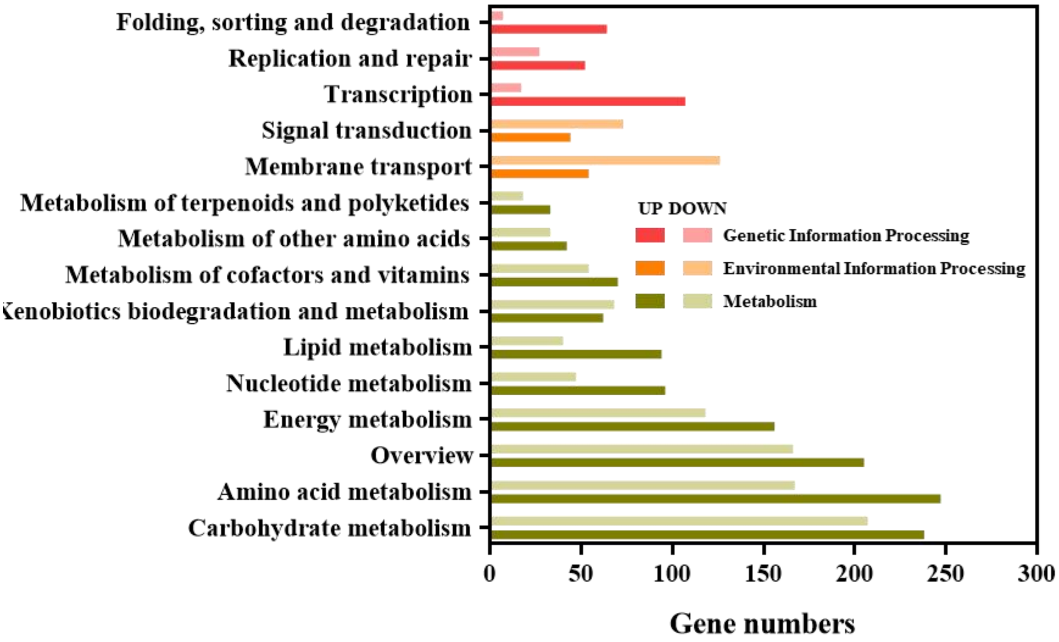


**Figure S3** Results of the KEGG classification analysis of differentially expressed genes between T1-THI and T1-CK. The dark-colored columns indicate the number of significantly upregulated genes in microbial culture H-1 with exogenous thiamine, while the light-colored columns indicate the number of significantly downregulated genes.

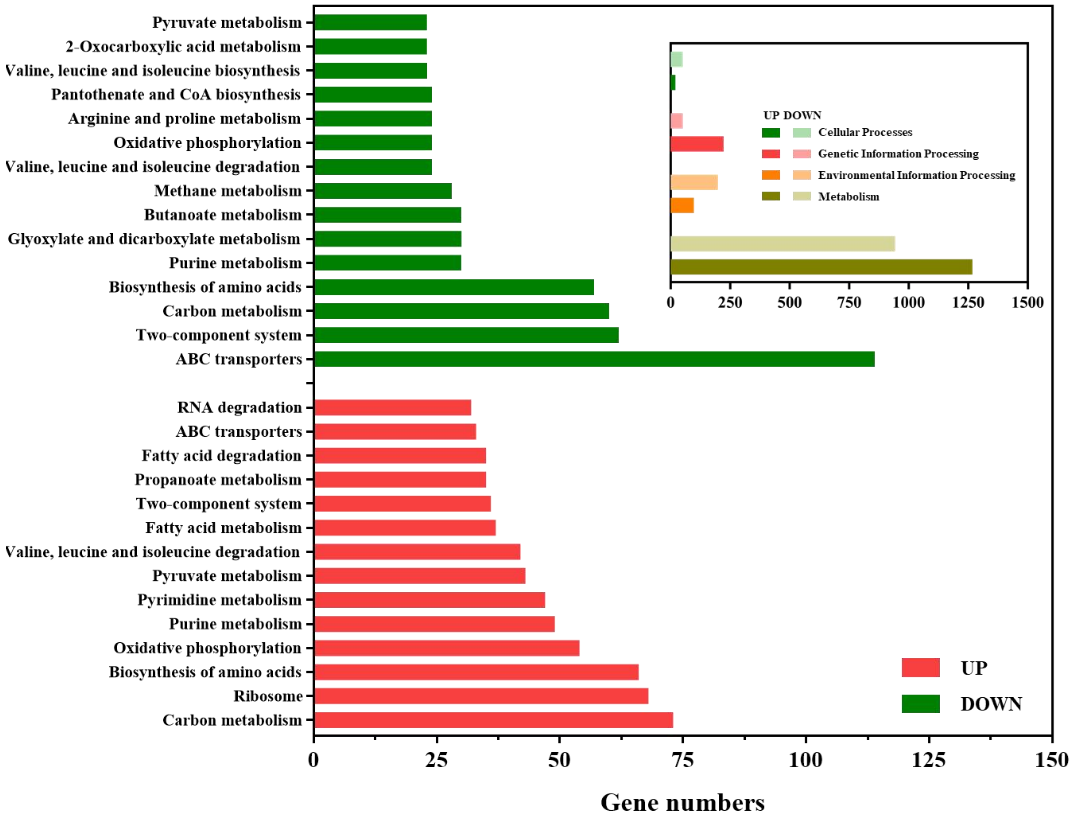


**Figure S4** Results of the KEGG classification analysis of differentially expressed genes (DEGs) between T1-THI and T1-CK. The number of specific upregulated (red bar) and downregulated (green bar) genes are indicated. The plot within the figure shows the number of DEGs involved in major metabolic pathways. The dark-colored columns indicate the number of significantly upregulated genes in microbial culture H-1 with exogenous thiamine, while the light-colored columns indicate the number of significantly downregulated genes.

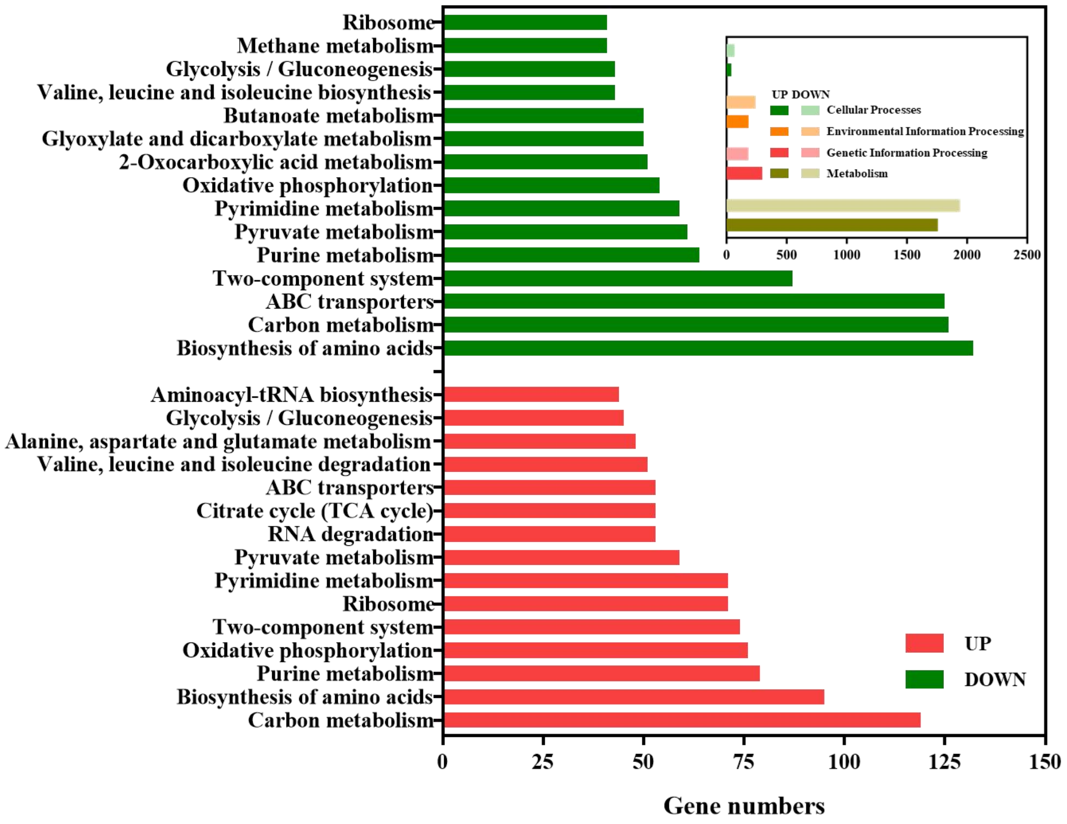


**Figure S5** Results of the KEGG classification analysis of differentially expressed genes (DEGs) between T40-THI and T40-CK. The number of specific upregulated (red bar) and downregulated (green bar) genes are indicated. The plot within the figure shows the number of DEGs involved in major metabolic pathways.

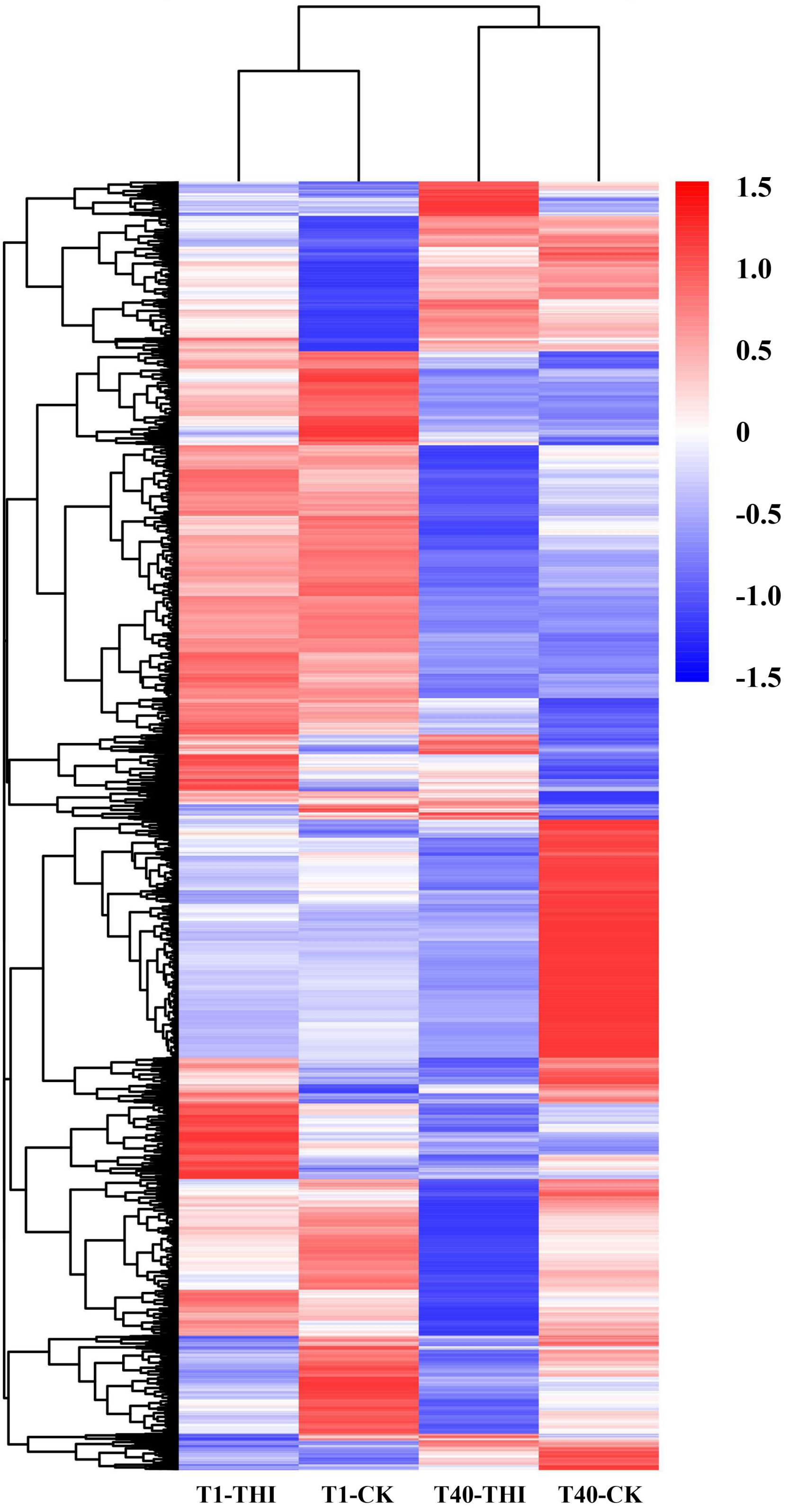


**Figure S6** Heatmap of differentially expressed genes (DEGs) in all groups. Each column on the horizontal axis represents one of the DEGs, and each row on the vertical axis represents one of the four groups. Expression levels are depicted with a color scale, in which shades of red represent higher expression and shades of blue represent lower expression.

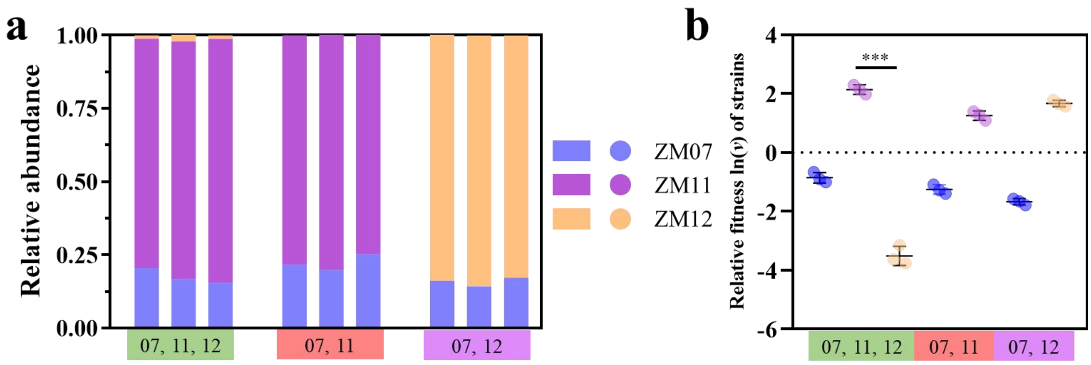


**Figure S7** Relative abundances (a) and relative fitness ln ($v$) (b) of ZM07, ZM11 and ZM12 in two-strain and three-strain systems at 48 h in the 3rd transfer. The $p$ value indicates statistical significance between ZM07, ZM11, and ZM12 determined using Student's $t$-test ($n = 3$, ***$p < 0.001$). Error bars represent the standard error of the mean.

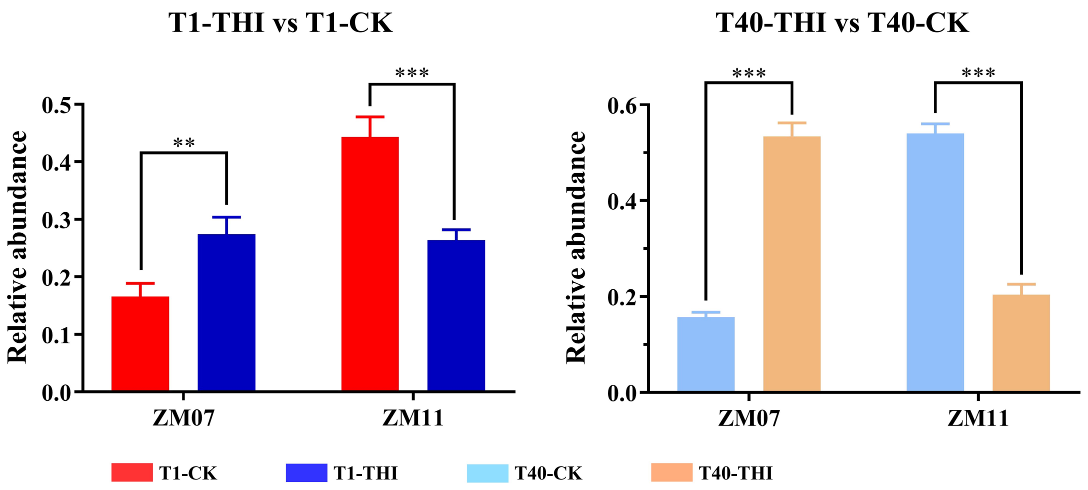


**Figure S8** The relative abundance of strains ZM07 and ZM11 in different groups. The *p* value indicates statistical significance determined using Student's *t*-test ($n = 12$, $^{**}p < 0.01$, $^{***}p < 0.001$). Error bars represent the standard error of the mean.

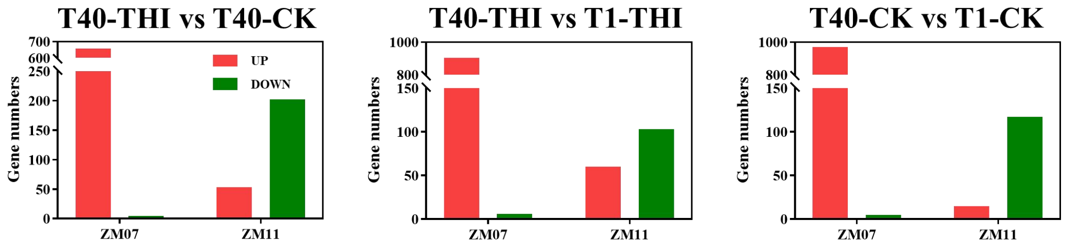


**Figure S9** Summary of differentially expressed genes of *Rhodococcus ruber* ZM07 and *Hydrogenophaga intermedia* ZM11 between different groups.

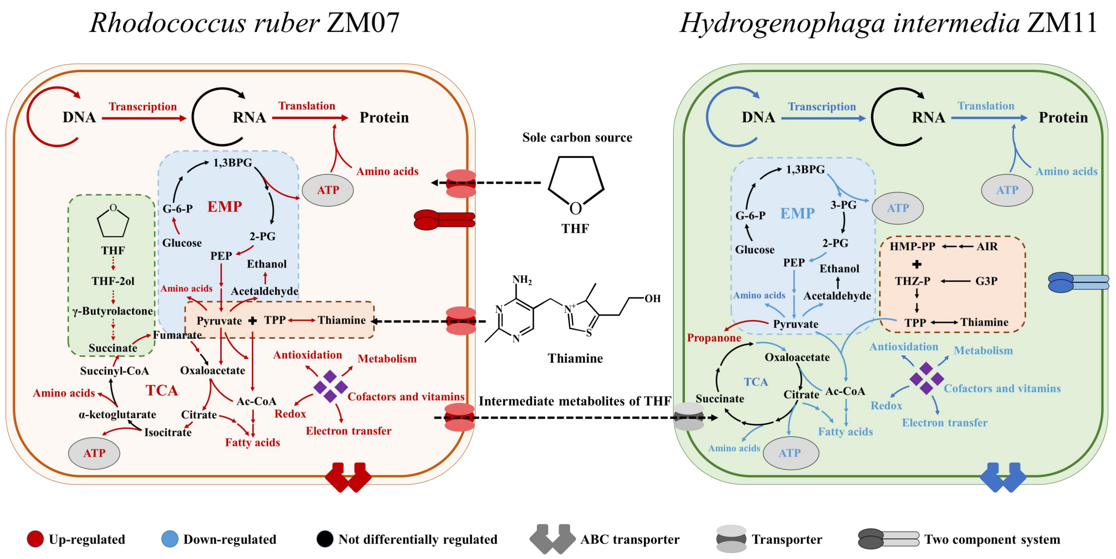


**Figure S10** The “seesaw mode” between thiamine auxotrophic ZM07 and the cooperator ZM11 after adding exogenous thiamine based on metatranscriptomic analyses.

## REFERENCES


1. Parales, R., Adamus, J., White, N., May, H. 1994. Degradation of 1, 4-dioxane by an actinomycete in pure culture. Appl Environ Microbiol 60:4527-4530. https://doi.org/10.1128/aem.60.12.4527-4530.1994.

2. Davis, D.H., Stanier, R., Doudoroff, M., Mandel, M. 1970. Taxonomic studies on some cram negative polarly flagellated “hydrogen bacteria” and related species. Arch Mikrobiol 70:1-13. https://doi.org/10.1007/bf00691056.

3. Lee, J.-J., Srinivasan, S., Kim, M.K. 2011. *Pigmentiphaga soli* sp. nov., a bacterium isolated from soil. J Microbiol 49:857-861. https://doi.org/10.1007/s12275-011-1375-8.

4. Reasoner, D.J., Geldreich, E. 1985. A new medium for the enumeration and subculture of bacteria from potable water. Appl Environ Microbiol 49:1-7. https://doi.org/10.1128/aem.49.1.1-7.1985.

5. Mortazavi, A., Williams, B.A., McCue, K., Schaeffer, L., Wold, B. 2008. Mapping and quantifying mammalian transcriptomes by RNA-Seq. Nat Methods 5:621-628. https://doi.org/10.1038/nmeth.1226.

6. Love, M.I., Huber, W., Anders, S. 2014. Moderated estimation of fold change and dispersion for RNA-seq data with DESeq2. Genome Biol 15:1-21. https://doi.org/10.1186/s13059-014-0550-8.

7. Kanehisa, M., Goto, S., Kawashima, S., Okuno, Y., Hattori, M. 2004. The KEGG resource for deciphering the genome. Nucleic Acids Res 32:277-280. https://doi.org/10.1093/nar/gkh063.
